# Trends in recorded deaths involving antipsychotics: The role of deprivation, ethnicity, and regional disparities

**DOI:** 10.1371/journal.pone.0349877

**Published:** 2026-06-12

**Authors:** Muhammad Umair Khan, Syed Shahzad Hasan

**Affiliations:** 1 Aston Pharmacy School, School of Medicine, Pharmacy and Biosciences, Aston University, Birmingham, United Kingdom; 2 School of Applied Sciences, University of Huddersfield, Huddersfield, United Kingdom; Fukuoka University, JAPAN

## Abstract

**Objective:**

The relationship between antipsychotic use and mortality remains uncertain, with evidence suggesting both potential risks and benefits. This study aimed to examine trends in recorded deaths involving antipsychotics in England and to explore regional, socioeconomic, and ethnic variation in these patterns.

**Methods:**

A population-level analysis of NHS prescription data (2015–2023) across seven regions of NHS England used linear regression and generalised additive models (GAMs) to explore associations between recorded deaths involving antipsychotics and deprivation, ethnic density, and regional variation.

**Results:**

Recorded deaths involving antipsychotics increased from 10.33 to 13.85 deaths per million prescription items between 2015 and 2023 (p < 0.01), with an annual increase of 3.56%. Regional analysis showed significant disparities, with the highest increase observed in London (9.99%, 95%CI: 9.50–10.48) and a decrease was observed in the Midlands (−2.06%, 95%CI: −2.71 – −1.41). Deprivation was significantly associated with increased mortality (p < 0.01), while regions with higher Asian density exhibited lower mortality rates (p = 0.01).

**Conclusions:**

Recorded deaths involving antipsychotics have increased over the past decade, with considerable regional disparities. Deprivation remains a key driver of mortality risk. While antipsychotics have well‑established clinical benefits in the management of severe mental illness, these findings highlight the need for strengthened prescribing safety, improved monitoring, and equity‑focused interventions to reduce preventable deaths.

## Introduction

Antipsychotic medications are integral to the management of severe mental health conditions, including schizophrenia, bipolar disorder, and treatment-resistant depression [1]. Their effectiveness in managing symptoms and preventing relapses is well-established in the literature [2–5]. However, concerns persist regarding their safety profile [6]. Adverse outcomes associated with antipsychotics include drug-induced movement disorders, metabolic dysfunction, and severe events such as toxicity and death [6]. These risks underscore the importance of continuously monitoring antipsychotic use and associated mortality trends.

Global evidence indicates an increasing use of antipsychotics [7], with a similar trend observed in the UK, particularly for second-generation antipsychotics [8]. Although prescribing patterns before 2020 were extensively studied, the COVID-19 pandemic introduced significant disruptions to mental health care delivery, such as reduced access to in-person services, heightened stress, and social isolation. These factors may have altered antipsychotic prescribing practices, but little is known about how these trends evolved after the pandemic’s acute phase. While antipsychotics are effective in managing symptoms and may contribute to reduced mortality through improved clinical stability and reduced risk of adverse outcomes, the overall impact of these medications on mortality remains uncertain. Some studies suggest a potential protective effect associated with treatment adherence, symptom control, and reduced risk of relapse [9,10], whereas others report increased mortality risk, potentially linked to adverse effects such as metabolic and cardiovascular complications [11,12]. Therefore, the relationship between antipsychotic prescribing patterns and mortality outcomes remains complex and not yet fully understood [13].

In the UK, studies have offered limited insights into this issue. For example, a study investigating mortality rates from 1992 to 2005 in children and adolescents showed an elevated standardised mortality ratio in patients exposed to antipsychotic use but concluded that it could be due to an underlying medical condition [14]. Another study showed that unintentional second-generation antipsychotic poisoning increased steadily from 1998 to 2019 [11]. However, a key gap in the literature is whether mortality rates changed independently of prescription counts or as a proportion of prescriptions issued. Investigating these dynamics is crucial for determining whether medication use is optimised or if gaps in prescribing or monitoring contribute to fatal outcomes. Variations in prescribing patterns, including differences in drug choice, dosing, initiation, and monitoring, may contribute to differences in mortality risk [15]. Understanding these variations across regions and population groups is therefore important for identifying potential inequalities in care and informing safer and more equitable prescribing practices.

The influence of deprivation and ethnicity on antipsychotic use is well-established in the literature [8]. Emerging evidence suggests that socio-demographic factors may also influence deaths involving antipsychotics [16,17]; however, this area remains underexplored. In particular, ethnic density—the concentration of ethnic groups within specific regions—may play a crucial role in shaping these outcomes, with some studies suggesting it may have some protective effect against psychoses, particularly in the context of the British Asian population, potentially through mechanisms such as increased social support and reduced discrimination [18]. These contextual factors may, in turn, influence patterns of service use, prescribing, and treatment engagement, which could be reflected in variation in deaths involving antipsychotics. Understanding these relationships is essential for addressing health inequities and guiding targeted interventions.

Regional disparities further complicate the dynamics of antipsychotic-related mortality. Existing evidence highlights variations in population demographics, healthcare resources, and deprivation across England [19]. These variations have been linked to differences in antipsychotic prescribing practices among demographic groups and areas of deprivation [8,20], potentially influencing mortality patterns. However, the extent and impact of these disparities in England remain unclear. Understanding these disparities is essential for informing prescribing practices and improving outcomes. Furthermore, the COVID-19 pandemic has further complicated these dynamics, potentially altering prescribing patterns, healthcare access, and mortality trends, making it crucial to understand its impact on antipsychotic-related mortality outcomes.

This study addresses these critical gaps by analysing trends in antipsychotic prescribing and recorded deaths involving antipsychotics in England from 2015 to 2023. It also explores the role of regional differences, deprivation and the impact of ethnic density on mortality outcomes in patients using antipsychotic medication.

## Materials and methods

### Data design and sources

This population-level observational study used publicly available national datasets. Prescription data were obtained from the NHS Business Services Authority (NHSBSA), which publishes monthly records of prescriptions issued in England and dispensed in community settings [21]. Prescriptions issued outside England or dispensed in other settings, such as hospitals and prisons, are excluded from these records. For medicines used in mental health, including antipsychotics, the NHSBSA compiles annual summaries presenting prescription item counts at national and integrated board (ICB) levels. Data on deaths involving antipsychotics in England were sourced from the UK Office for National Statistics (ONS) [22]. The NHSBSA and ONS mortality data are widely used and cited in the literature [23–25]. Ethnicity data were obtained from the ONS based on the 2021 Census, which provides detailed demographic characteristics for England and Wales [26]. Deprivation indices were obtained from the English Indices of Deprivation 2019, a composite area-level measure of socioeconomic disadvantage, which includes measures across multiple domains such as income, employment, education, and health [27]. The deprivation score for England and its regions is publicly available through Fingertips, a large public health data collection. These indices are widely used to evaluate socioeconomic inequalities at regional and local levels [28,29]. This study is reported following the RECORD checklist for reporting observational studies (S1 Table).

### Data extraction and processing

Prescription data were extracted from the NHSBSA-compiled summaries from 2015 to 2023. The extracted data included all dispensed antipsychotics identified using the British National Formulary section code 0402. These data were initially extracted at an ICB level, with 42 ICBs then grouped into seven NHS England regions (East of England, London, Midlands, North-East and Yorkshire, North-West, South-East, and South-West). The extracted data comprised each region’s annual number of prescription items over the study period. A prescription item is defined as a single prescription for a particular drug. Mortality data were extracted from the ONS records of deaths related to drug poisoning in England and Wales, which provides publicly accessible aggregated mortality data. Specifically, we extracted annual counts of deaths involving antipsychotic drugs as reported in the ONS tables. The ONS methodology, including the definition of drug poisoning deaths and the identification of substances mentioned on death certificates, is fully documented within its publication [22]. These data were available for England and its regions, allowing for national and regional analyses of antipsychotic-related deaths.

Ethnicity data were extracted from the ONS 2021 Census for people with Asian backgrounds. In the context of England’s diverse population, ethnic density may influence not only the incidence and course of psychotic disorders but also treatment patterns and associated outcomes, including mortality [30,31]. The inclusion of the Asian population as a predictor was informed by prior evidence on the protective effects of ethnic density in relation to mortality in severe mental illness [31]. Whilst the protective effects against psychoses are relatively more established in African populations [30], emerging evidence from studies in Asian populations suggests similar patterns [18]. However, further research is required to confirm the consistency and generalisability of these findings. We divided the total number of Asian residents within each ICB by the overall ICB population to generate a percentage measure of ethnic density, with a higher percentage indicating a higher relative proportion of Asian residents.

### Operational definition

Deaths involving antipsychotics were defined as any drug poisoning death (e.g., accidental, intentional) as described by the International Classiﬁcation of Diseases Codes (S2 Table) where an antipsychotic (S3 Table) was mentioned on the death certiﬁcate, with or without concomitant mention of alcohol or other substances. This definition is restricted to drug poisoning deaths and does not include deaths related to chronic physical complications associated with long-term antipsychotic use (e.g., cardiovascular or metabolic causes).

### Data analyses

Regional annual prescription data were aggregated to calculate the national-level prescription count for each year. Linear regression was used to model the association between antipsychotic prescription and deaths throughout the study period. Analyses were stratified by NHS England regions, enabling comparisons of mortality trends across seven regions. The mean annual change in deaths involving antipsychotics in England and its regions was calculated by dividing the regression coefficient by the baseline prescription count (2015). To assess whether regional changes were consistent over time, the mean annual change was further stratified by two distinct periods: 2015–2019 and 2020–2023.

Linear regression models were also employed to explore associations between mortality rates per million antipsychotic prescriptions, deprivation and ethnicity (Asian). All variables were treated as continuous. Generalised Additive Models (GAMs) accounted for potential non-linear relationships. GAMs allowed for smooth terms, accounting for a non-linear complex association between the predictor variables (Asian, deprivation) and the response variable (mortality rate per million prescriptions). The significance of smooth and linear terms was assessed using the F-test and t-test. Overall model fit was evaluated using an adjusted R-squared, generalised cross-validation score, Akaike information criterion, and Bayesian information criterion. Statistical significance was determined using a 95% confidence interval and a p-value threshold of 0.05. All analyses were conducted using Microsoft Excel and R Foundation for Statistical Computing, version 4.3.1.

### Ethics statement

Not applicable. Ethical approval was not required for this study because we used the NHSBSA database, which is a free, open-access database and contains anonymised population-level information.

## Results

**Fig 1:**
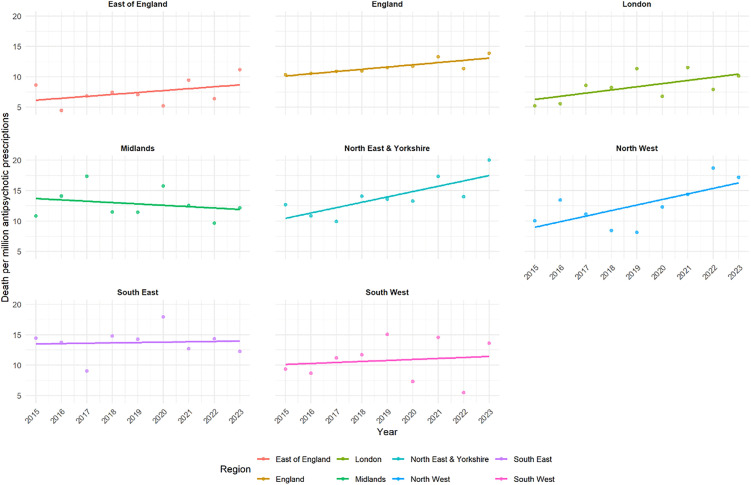
Deaths per million antipsychotic prescriptions in England and its regions.

The study showed a consistent rise in recorded deaths involving antipsychotics in England across the study period (2015–2023). The death rate increased from 10.33 deaths in 2015 to 13.85 deaths per million antipsychotic prescription items in 2023. The linear regression analysis at the national level showed a statistically significant positive coefficient, indicating an increasing upward trend (p < 0.01) (S4 Table, S5 Table, and Fig 1). Regional analysis showed substantial variations. All regions except the Midlands showed an overall increase in the number of deaths per million antipsychotic prescription items. However, statistical significance was only achieved in the North-East & Yorkshire and North-West regions (p < 0.05) (S5 Table and Fig 1).

**Fig 2:**
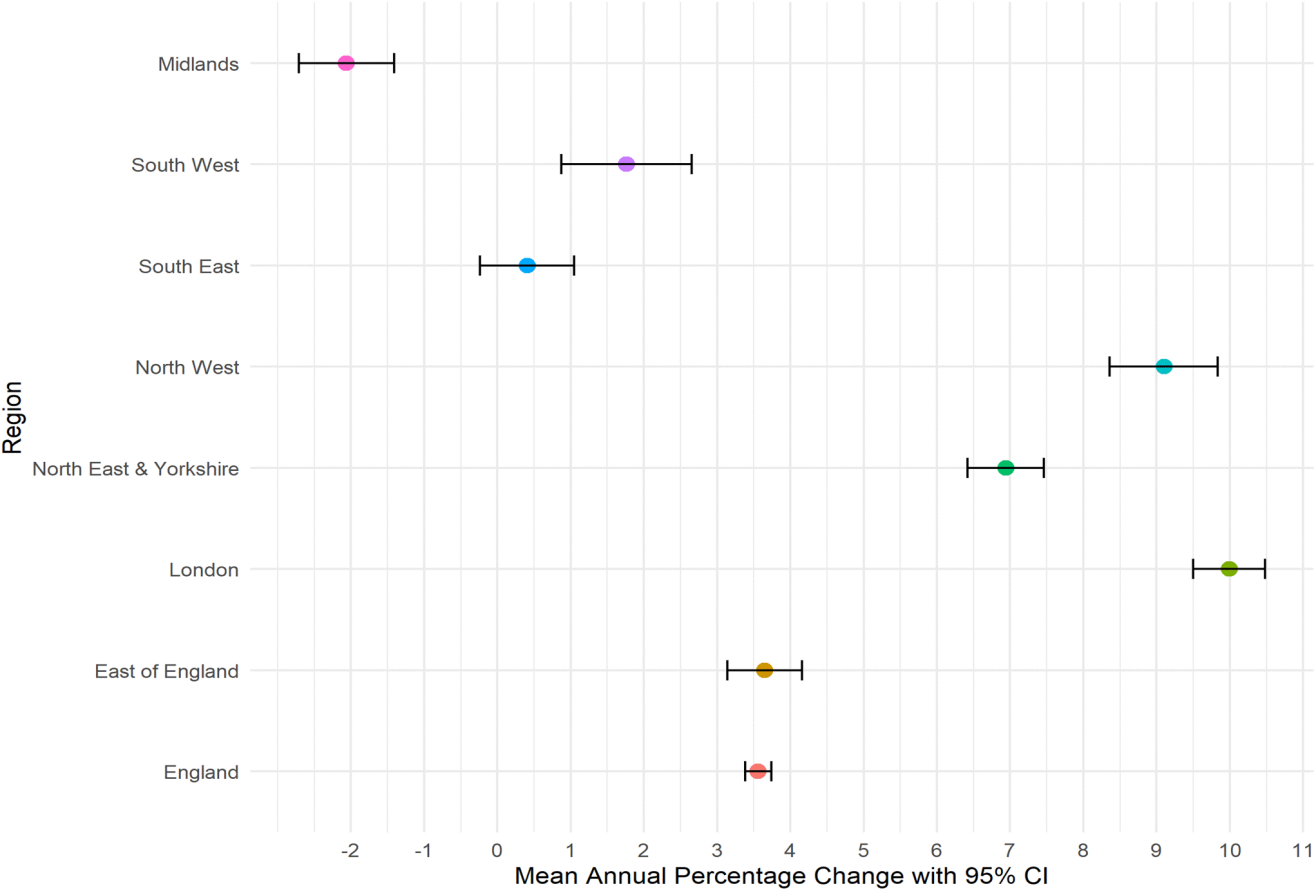
Mean annual change in deaths per million antipsychotic prescriptions.

The study also explored the changes in the death rate in England and its regions across the study period. Nationally, the number of deaths per million prescription items increased at 3.56% per annum (95%CI = 3.38–3.74). The highest increase in the rate of deaths was observed in the London region (9.99%, 95%CI = 9.50–10.48), followed by North-West (9.10%, 95%CI = 8.36–9.83) and North-East & Yorkshire (6.94%, 95%CI = 6.42–7.46). In contrast, a decrease in the death rates was observed in the Midlands (−2.06%, 95%CI = −2.71 – −1.41) (Fig 2 and S5 Table).

The study also found some differences in the rate of deaths between the two sub-periods (2015–2019 & 2020–2023). It was also observed that the death rates in North-West (13.37% vs −8.81%) and North-East & Yorkshire (12.81% vs 3.96%) were relatively higher in the latter period compared to the other regions, where no significant increase was observed between the two periods (S1 Fig). At the national level, the annual increase in death rate rose from 2.67% in 2015−19 to 3.73% in 2020−23.

**Fig 3:**
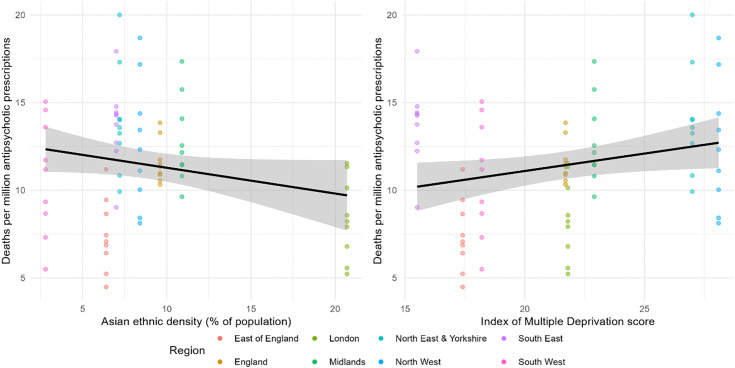
Linear analysis of Asian ethnic population proportion, deprivation, and rates of deaths involving antipsychotics.

**Fig 4:**
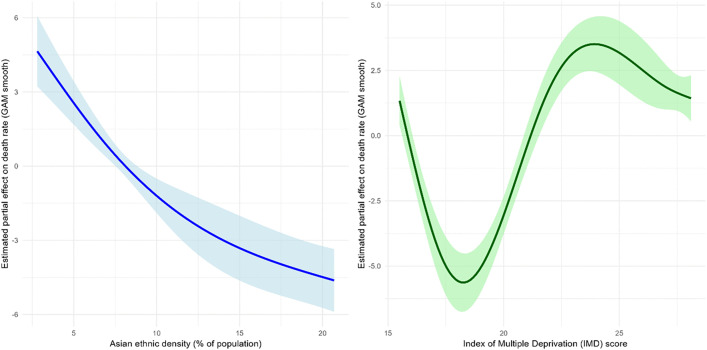
Smooth term analysis of Asian ethnic population proportion, deprivation, and rates of deaths involving antipsychotics.

Deprivation was found to be significantly associated with deaths involving antipsychotics in the linear regression model (p < 0.01), with regions with higher levels of deprivation showing higher mortality rates. Similarly, ethnicity was also a significant determinant, with regions with a higher density of Asian population being associated with reduced mortality rates (p < 0.05) (S6 Table and Fig 3). Smooth term analysis showed similar trends, with both deprivation and ethnicity being found to be significantly associated with mortality rates (p < 0.01). However, the association between mortality rates and deprivation was found to be non-linear (EDF = 3.90), suggesting a complex relationship that may reflect the influence of additional unmeasured social and healthcare-related factors across levels of deprivation (S7 Table and Fig 4).

## Discussion

This descriptive, observational study evaluates patterns of recorded deaths involving antipsychotics in England, exploring regional differences and the role of deprivation and ethnic variation, without aiming to identify causal mechanisms underlying these trends. Our findings indicate a consistent rise in mortality, increasing from 10.33 to 13.85 deaths per million antipsychotic prescription items between 2015 and 2023. These findings align with previous research [32], which reported a higher adjusted risk of death with first-generation antipsychotics in schizophrenia and increased mortality with oral formulations compared to long-acting injectables for both first- and second-generation antipsychotics. Several mechanisms have been proposed in the literature that may potentially contribute to these patterns, including dose-related cardiotoxic and central nervous system effects [33,34]. However, it is important to note that these findings specifically reflect trends in drug poisoning deaths involving antipsychotics and should be interpreted in this context, rather than as representing overall mortality associated with long-term antipsychotic use. Furthermore, studies using linked individual-level datasets are needed to examine cause-specific mortality and differences between antipsychotic classes (e.g., first- versus second-generation agents), which may help clarify whether observed patterns are driven by acute toxicity, long-term adverse effects, or underlying differences in patient populations and care. This study is best interpreted as examining contextual vulnerability to acute toxic events among people exposed to antipsychotics, rather than risk intrinsic to antipsychotics themselves.

Antipsychotics are a cornerstone in the management of severe mental illness, with well-established efficacy in reducing psychotic symptoms and relapse [9,10]. However, the relationship between their use and mortality is complex and may be influenced by prescribing patterns, particularly during the initial phase of treatment. While these factors were not directly examined in this study, previous studies suggest that dosing may influence deaths involving antipsychotics, with the greatest risk of death occurring in the early stages of therapy and with higher doses, particularly in the older population [35]. Arai et al. found that new users and those with shorter treatment durations had a higher mortality risk [36], while Ray et al. reported a dose-dependent increase in sudden cardiac death risk across both first- and second-generation antipsychotics [37]. Similarly, another study identified higher quetiapine doses as a significant predictor of increased mortality [38]. Although this study does not have access to longitudinal prescribing data to confirm changes in dosing over time, the observed increase in mortality may plausibly reflect a combination of factors, including changes in prescribing practices over time, such as increased polypharmacy, as well as differences in service capacity and monitoring intensity across regions, which may influence both prescribing safety and the timely management of adverse effects [15].

In addition, it is important to consider that observed mortality patterns may also reflect multiple contributing factors, particularly in older adults, such as patient-level vulnerability, treatment initiation, increased sensitivity to side-effects, rather than prescribing intensity alone [36,38]. While this study describes trends in deaths involving antipsychotics, future research could further delineate how these risks evolve over the course of therapy. Specifically, further research is needed to determine whether the elevated mortality risk is higher during the initial treatment period or continues with long-term use.

Our study demonstrates significant regional and socioeconomic variations in deaths involving antipsychotics. These disparities may potentially stem from differences in healthcare access, prescribing practices (e.g., choice of antipsychotic, dosing, treatment duration) and levels of socioeconomic deprivation, all of which may influence treatment adherence, medication monitoring, and overall health outcomes. In addition, regional differences in the prevalence and severity of severe mental illness may also contribute to the observed variation, as populations with higher underlying disease burden are likely to have both increased antipsychotic prescribing and higher baseline mortality risk. These prescribing variations, when considered alongside regional and socioeconomic disparities, may help explain differences in deaths involving antipsychotics and highlight opportunities to improve prescribing practices and monitoring in higher-risk populations [15]. Such disparities highlight the need for further investigation into the factors driving inequities in antipsychotic prescribing, particularly in populations at higher risk of adverse effects.

Our findings on deprivation are consistent with existing literature, which highlights the significant impact of socioeconomic disadvantage on deaths involving antipsychotics. A study found that lower socioeconomic status was associated with delays in diagnosis, reduced access to specialist care, and higher doses of antipsychotic medications, all of which contribute to increased mortality risk [39]. Similarly, another study reported that individuals from deprived backgrounds face greater barriers to receiving optimal mental health treatment, further exacerbating health disparities [40]. These findings underscore the need for targeted interventions to reduce socioeconomic inequalities and improve access to safer and more effective treatments.

Ethnicity is another key factor that may potentially influence deaths involving antipsychotics, playing a role through pharmacokinetic and genetic variations, clinical differences in drug response and adverse effects, and social determinants such as ethnic density. Differences in drug metabolism, particularly variations in CYP2D6 enzyme activity, influence antipsychotic clearance and efficacy. A review found that certain Asian populations have a higher prevalence of CYP2D6 polymorphisms, leading to slower metabolism and increased sensitivity to antipsychotics like clozapine, which may necessitate lower dosing to reduce adverse effects [41]. Clinically, ethnic differences in response to antipsychotics have been observed, with studies reporting a higher risk of extrapyramidal side effects in minority populations compared to Caucasians, though the role of confounding factors remains unclear [42]. Given the heterogeneity within the ethnic and racial classifications and the limitations in accurately measuring genetic variation, the role of ethnicity in prescribing disparities remains an area of debate and requires further investigation [43,44]. However, it is important to note that such pharmacogenetic factors are unlikely to be directly observable in routine mortality surveillance data and may influence deaths involving antipsychotics only indirectly, for example, through dosing decisions, drug interactions, or patterns of monitoring, rather than acting as proximate causes of acute poisoning.

Our findings suggest a protective effect of ethnic density at a regional level, aligning with the ethnic density hypothesis. Higher ethnic density has been associated with improved mental health outcomes, potentially due to stronger social support networks, reduced discrimination, and better engagement with healthcare services [45]. This may have implications for deaths involving antipsychotics through several pathways. Improved engagement with healthcare services may facilitate earlier intervention, more consistent follow-up, and closer monitoring for adverse effects, including metabolic and cardiovascular complications associated with antipsychotic use [18,31]. This, in turn, may support more appropriate prescribing, timely dose adjustments, and improved adherence, which could reduce the risk of adverse outcomes.

Our findings on ethnic density may have implications for the development of targeted mental health interventions, particularly in regions with diverse populations. Ensuring equitable access to culturally appropriate services may enhance treatment adherence and outcomes, especially in areas with higher ethnic density. The intersection of ethnic density, genetic predisposition, and clinical response to antipsychotic treatment underscores the need for a more personalised and equitable approach to prescribing these medications. Therefore, a cautious and personalised approach to treatment should be encouraged, considering the adverse effects and variation in prescribing and cultural beliefs and practices. This approach is consistent with evidence supporting personalised prescribing frameworks that integrate individual risk profiles and side-effect burden to guide antipsychotic selection and monitoring [46].

The COVID-19 pandemic has led to an increased use of antipsychotic medications, widened disparities across populations, and exacerbated cultural factors affecting medication adherence, particularly impacting women, ethnic minorities, and those with chronic illnesses [47,48]. A Danish nationwide registry study found an elevated risk of severe infection outcomes, hospitalisation, and death in patients using antipsychotics during the pandemic [49]. Similarly, in a retrospective cohort study using the OpenSAFELY database, the researchers observed a spike in new prescriptions for patients with dementia and in care homes, correlating with COVID-19 lockdowns [50]. Interestingly, while public health measures initially resulted in a decline in the use of antipsychotics, overall use of antipsychotics remained stable during the pandemic [51]. Importantly, pandemic‑related factors, such as social isolation, reduced community support, economic insecurity, and barriers to healthcare access, may have increased vulnerability to acute toxic events, particularly in more deprived and ethnically diverse populations [52]. These findings suggest that external public health crises may further widen the gaps in prescribing disparities, necessitating further research to identify and address these inequities in mental health care delivery, particularly the use of antipsychotics.

### Strengths and limitations

The strength of this study lies in the use of robust national data sources such as NHS prescription numbers in millions, deaths involving antipsychotics statistics, and socioeconomic data from government sources that have been widely used in the literature. A key strength of this study is the standardisation of mortality rates using deaths per million antipsychotic prescription items, which partially accounts for temporal changes in prescribing volume and allows for more meaningful comparison of trends over time. Another strength of this study is its ability to highlight regional disparities, including variations linked to deprivation and ethnicity, in deaths involving antipsychotics across England, providing valuable information for targeted interventions, healthcare practice and policy development. Furthermore, the extended time period covered by this study is a key strength, as it allows for the exploration of potential differences in deaths involving antipsychotics before and after the COVID-19 pandemic, offering critical insights into evolving patterns and outcomes.

One major limitation is that these datasets are population-level, lacking patient-level information or parameters. The patient-level parameters could be useful in adjusting for confounding effects, such as age, gender, and diagnosis, when measuring the association between exposures and outcomes. As a result, the analyses are ecological in nature and do not allow inference at the individual patient level. Despite this, population-level data are important in studying trends across large populations, facilitating policy development, informed decision-making, and economic and social impact assessment, allowing for a holistic understanding of challenges beyond individual cases. Moreover, these findings offer a strong foundation for future research to explore underlying mechanisms in greater detail and address potential confounding factors more precisely.

A further important limitation is that the outcome measure is based on any mention of an antipsychotic on the death certificate, which does not establish causality or confirm that antipsychotics directly contributed to death. This approach may therefore overestimate the extent to which antipsychotics are implicated in mortality, as their presence may reflect co-prescribing, past exposure, or incidental mention rather than a causal role. However, this definition is consistent with routinely reported national mortality statistics and allows for population-level surveillance of potential safety signals.

An additional important limitation is the potential for reverse causation and confounding by indication. Antipsychotics are more likely to be prescribed to individuals with severe mental illness, such as schizophrenia, which is itself associated with increased all-cause mortality. Therefore, the observed association between antipsychotic prescribing and mortality may, in part, reflect underlying disease severity, regional variation in prevalence or severity, and higher baseline risk in the treated population rather than a direct effect of antipsychotic exposure. Furthermore, the aggregated data do not allow assessment of cause-specific mortality or differences between antipsychotic classes, limiting insight into underlying mechanisms. This limitation is inherent to ecological analyses using aggregated prescribing and mortality data.

Prescription data were derived from community-dispensed medications and may not fully capture antipsychotic dispensed in inpatient or secondary care settings, potentially underrepresenting individuals with more severe illness. Furthermore, this data reflects medications dispensed, not actual consumption. It is possible that some individuals did not adhere to prescribed treatments after collecting their medications from the pharmacy. Additionally, while this study focused on socio-demographic factors like ethnicity and deprivation, these may not fully account for the variations in prescribing practices and deaths involving antipsychotics observed across England.

The concept of ethnic density has been traditionally applied at smaller geographic units such as local neighbourhoods or Lower Layer Super Output Areas (LSOAs), where local social cohesion and reduced discrimination may contribute to improved mental health outcomes. Furthermore, temporal trend analyses stratified by deprivation and ethnic composition were not possible, as these variables were available as relatively time-invariant area-level measures rather than longitudinal exposures. While our analysis examines ethnic density at the NHS regional level, which is broader, the observed protective effect may still reflect underlying structural and healthcare-related factors that warrant further investigation at finer scales.

### Implications for practice, policy and research

Given the observed variations in deaths involving antipsychotics, clinicians should consider patient-specific factors, including ethnicity, socioeconomic status, and regional healthcare resources, when prescribing antipsychotic medications. This could help mitigate risks associated with antipsychotic use. Furthermore, continuous monitoring, especially during the initial stages of treatment and at higher doses, is recommended to minimise mortality risks. Deprescribing antipsychotics and non-pharmacological therapy may also be considered when appropriate to mitigate risk.

The regional disparities observed in deaths involving antipsychotics underline the need for targeted policy interventions that address variations in healthcare access, prescribing practices, and socio-economic conditions. Policymakers should prioritise reducing these regional inequities by ensuring that healthcare services are equally distributed across different regions, with a specific focus on areas with higher mortality rates. Policies aimed at improving social support networks, especially in deprived areas and ethnic minority communities, could reduce the adverse effects of antipsychotic medications and improve overall mental health outcomes.

Future research should further investigate the role of ethnic density, genetic factors, and regional disparities in antipsychotic prescribing and related mortality, with a focus on the underlying mechanisms driving these disparities. Additionally, research could explore how clinician decision-making influences prescribing practices, especially across different socioeconomic and ethnic groups. There is also a critical need to examine patient perspectives on medication access, adherence, and cultural attitudes toward mental health treatment, as these factors play a significant role in the clinical decision-making process. Furthermore, exploring systemic factors such as regional policies, funding allocation, and clinicians’ training is essential to understanding how these factors contribute to inequalities in prescribing patterns.

## Conclusions

This study provides insights into regional and socioeconomic disparities in recorded deaths involving antipsychotics in England, highlighting an overall increase in deaths over the study period. Substantial regional variation was also observed, including a decrease in the Midlands, highlighting heterogeneity in trends across England. The findings also show differences in mortality rates across areas defined by socioeconomic deprivation and ethnic population composition, with variation observed across regions. The results underscore the need for a more tailored and equitable approach to prescribing antipsychotics, considering clinical, social, and genetic factors. Addressing these regional and socio-demographic disparities could lead to more effective treatment strategies and improved patient outcomes, particularly for vulnerable populations. Further research is needed to explore the underlying mechanisms of these disparities, including clinician decision-making, and to examine whether temporal increases in drug-poisoning deaths involving antipsychotics vary across sociodemographic subgroups, using more granular longitudinal data. This would help to develop targeted interventions that reduce inequalities in mental health care, ultimately promoting better health outcomes across diverse populations.

## Supporting information

S1 Table The RECORD statement – checklist of items, extended from the STROBE statement, that should be reported in observational studies using routinely collected health data. (DOCX)

S2 Table ICD-10 codes for deaths involving drug use. (DOCX)

S3 Table List of antipsychotics. (DOCX)

S4 Table Antipsychotics prescription count across the study period. (DOCX)

S5 Table Regression analysis of deaths per million antipsychotic prescriptions in England and its regions. (DOCX)

S6 Table Linear regression analysis. (DOCX)

S7 Table Smooth term analysis. (DOCX)

S1 Fig Mean annual change in deaths per million antipsychotic prescriptions by region and period. (DOCX)
