# Supplementary material for: Trends in recorded deaths involving antipsychotics: The role of deprivation, ethnicity, and regional disparities
Source: PLoS One. 2026 Jun 12;21(6):e0349877. doi: 10.1371/journal.pone.0349877 (PMC13262819; doi:10.1371/journal.pone.0349877)
Supplement: S2 Table — (DOCX) [file pone.0349877.s002.docx]

**Table S2: ICD-10 codes for deaths involving drug use**

| **ICD-10 codes** | **Description** |
| --- | --- |
| F11–F16, F18–F19 | Mental and behavioural disorders due to drug use (excluding alcohol and tobacco) |
| X40–X44 | Accidental poisoning by drugs, medicaments and biological substances |
| X60–X64 | Intentional self-poisoning by drugs, medicaments and biological substances |
| Y10–Y14 | Poisoning by drugs, medicaments and biological substances, undetermined intent |
| X85 | Assault by drugs, medicaments and biological substances |
