# Supplementary material for: Trends in recorded deaths involving antipsychotics: The role of deprivation, ethnicity, and regional disparities
Source: PLoS One. 2026 Jun 12;21(6):e0349877. doi: 10.1371/journal.pone.0349877 (PMC13262819; doi:10.1371/journal.pone.0349877)
Supplement: S3 Table — (DOCX) [file pone.0349877.s003.docx]

**Table S3: List of antipsychotics**

| **Typical** | **Atypical** | **Antipsychotic depot injections** |
| --- | --- | --- |
| - Benperidol - Chlorpromazine - Fluphenazine decanoate - Flupentixol - Haloperidol - Levomepromazine - Pericyazine - Perphenazine - Pimozide - Prochlorperazine - Promazine - Sulpiride - Trifluoperazine - Zuclopenthixol | - Amisulpride - Aripiprazole - Asenapine - Cariprazine - Clozapine - Lurasidone hydrochloride - Olanzapine - Paliperidone - Quetiapine - Risperidone | - Aripiprazole - Flupentixol decanoate - Fluphenazine decanoate - Haloperidol - Olanzapine embonate - Paliperidone - Pipotiazine palmitate - Risperidone - Zuclopenthixol decanoate |
