# Supplementary material for: Trends in recorded deaths involving antipsychotics: The role of deprivation, ethnicity, and regional disparities
Source: PLoS One. 2026 Jun 12;21(6):e0349877. doi: 10.1371/journal.pone.0349877 (PMC13262819; doi:10.1371/journal.pone.0349877)
Supplement: S4 Table — (DOCX) [file pone.0349877.s004.docx]

**Table S4: Antipsychotics prescription count (numbers) across the study period**

| **Region** | **2015** | **2016** | **2017** | **2018** | **2019** | **2020** | **2021** | **2022** | **2023** |
| --- | --- | --- | --- | --- | --- | --- | --- | --- | --- |
| England | 9,292,990 | 9,858,186 | 10,283,818 | 10,662,139 | 11,129,262 | 11,584,079 | 11,812,779 | 11,992,120 | 12,197,908 |
| East of England | 1,040,921 | 1,116,702 | 1,165,674 | 1,211,058 | 1,273,790 | 1,337,948 | 1,375,099 | 1,403,501 | 1,430,415 |
| London | 1,341,983 | 1,439,795 | 1,516,468 | 1,581,037 | 1,676,118 | 1,767,055 | 1,821,609 | 1,893,957 | 1,972,623 |
| North East & Yorkshire | 1,577,522 | 1,659,950 | 1,713,164 | 1,778,035 | 1,841,258 | 1,886,296 | 1,906,856 | 1,927,480 | 1,948,935 |
| North West | 1,494,960 | 1,562,125 | 1,620,384 | 1,662,694 | 1,723,263 | 1,786,136 | 1,808,637 | 1,818,986 | 1,804,618 |
| South East | 1,316,533 | 1,381,768 | 1,440,554 | 1,488,440 | 1,542,162 | 1,617,090 | 1,652,747 | 1,673,432 | 1,715,594 |
| South West | 856,211 | 922,883 | 983,326 | 1,023,977 | 1,062,715 | 1,093,908 | 1,097,113 | 1,093,684 | 1,102,995 |
| Midlands | 1,664,860 | 1,774,963 | 1,844,248 | 1,916,898 | 2,009,956 | 2,095,646 | 2,150,718 | 2,181,080 | 2,222,728 |
