# Supplementary material for: Trends in recorded deaths involving antipsychotics: The role of deprivation, ethnicity, and regional disparities
Source: PLoS One. 2026 Jun 12;21(6):e0349877. doi: 10.1371/journal.pone.0349877 (PMC13262819; doi:10.1371/journal.pone.0349877)
Supplement: S5 Table — (DOCX) [file pone.0349877.s005.docx]

| **Table S5: Regression analysis of deaths per million antipsychotic prescriptions in England and its regions** | | | | | | | | | | | | |
| --- | --- | --- | --- | --- | --- | --- | --- | --- | --- | --- | --- | --- |
| **Region** | **Deaths per million antipsychotic prescriptions** | | | | | | | | | **Coefficient** | **Std. Error** | **P-value** |
|  | **2015** | **2016** | **2017** | **2018** | **2019** | **2020** | **2021** | **2022** | **2023** |  |  |  |
| England | 10.33 | 10.55 | 10.89 | 10.97 | 11.50 | 11.74 | 13.29 | 11.34 | 13.85 | 0.37 | 0.09 | 0.01 |
| East of England | 8.65 | 4.48 | 6.86 | 7.43 | 7.07 | 5.23 | 9.45 | 6.41 | 11.19 | 0.32 | 0.26 | 0.27 |
| London | 5.22 | 5.56 | 8.57 | 8.22 | 11.34 | 6.79 | 11.53 | 7.92 | 10.14 | 0.52 | 0.25 | 0.08 |
| North East & Yorkshire | 12.68 | 10.84 | 9.92 | 14.06 | 13.58 | 13.25 | 17.31 | 14.01 | 20.01 | 0.88 | 0.27 | 0.01 |
| North West | 10.03 | 13.44 | 11.11 | 8.42 | 8.12 | 12.32 | 14.38 | 18.69 | 17.18 | 0.91 | 0.37 | 0.04 |
| South East | 14.43 | 13.75 | 9.02 | 14.78 | 14.27 | 17.93 | 12.71 | 14.34 | 12.24 | 0.06 | 0.33 | 0.86 |
| South West | 9.34 | 8.67 | 11.19 | 11.72 | 15.06 | 7.31 | 14.58 | 5.49 | 13.60 | 0.16 | 0.45 | 0.73 |
| Midlands | 10.81 | 14.08 | 17.35 | 11.48 | 11.44 | 15.75 | 12.55 | 9.63 | 12.15 | -0.22 | 0.33 | 0.52 |
