# Supplementary material for: Trends in recorded deaths involving antipsychotics: The role of deprivation, ethnicity, and regional disparities
Source: PLoS One. 2026 Jun 12;21(6):e0349877. doi: 10.1371/journal.pone.0349877 (PMC13262819; doi:10.1371/journal.pone.0349877)
Supplement: S6 Table — (DOCX) [file pone.0349877.s006.docx]

**Table S6: Linear regression analysis**

| **Factor** | **Regression Coefficient** | **Standard Error** | **T-Value** | **P-Value** |
| --- | --- | --- | --- | --- |
| (Intercept) | 7.81 | 2.01 | 3.88 | <0.01 |
| Asian | -0.19 | 0.07 | -2.44 | 0.01 |
| Deprivation | 0.24 | 0.09 | 2.67 | <0.01 |

Residual standard error = 3.21

Multiple R-squared = 0.13

Adjusted R-squared = 0.11

F-statistic = 5.39

P-value < 0.01
