# Supplementary material for: Trends in recorded deaths involving antipsychotics: The role of deprivation, ethnicity, and regional disparities
Source: PLoS One. 2026 Jun 12;21(6):e0349877. doi: 10.1371/journal.pone.0349877 (PMC13262819; doi:10.1371/journal.pone.0349877)
Supplement: S7 Table — (DOCX) [file pone.0349877.s007.docx]

**Table S7: Smooth term analysis**

| **Factor** | **Estimate** | **Standard Error** | **T-Value** | **P-Value** |
| --- | --- | --- | --- | --- |
| (Intercept) | 11.41 | 0.31 | 36.47 | <0.01 |
| **Smooth term** | **EDF** | **Ref. EDF** | **F-Value** | **P-Value** |
| Asian | 1.86 | 2.14 | 6.27 | <0.01 |
| Deprivation | 3.90 | 3.98 | 8.38 | <0.01 |

Adjusted R-squared = 0.39

Deviance explained = 44.2%

GCV = 7.77

Scale est. = 7.04
