# Supplementary material for: Trends in recorded deaths involving antipsychotics: The role of deprivation, ethnicity, and regional disparities
Source: PLoS One. 2026 Jun 12;21(6):e0349877. doi: 10.1371/journal.pone.0349877 (PMC13262819; doi:10.1371/journal.pone.0349877)
Supplement: S1 Fig — (DOCX) [file pone.0349877.s008.docx]

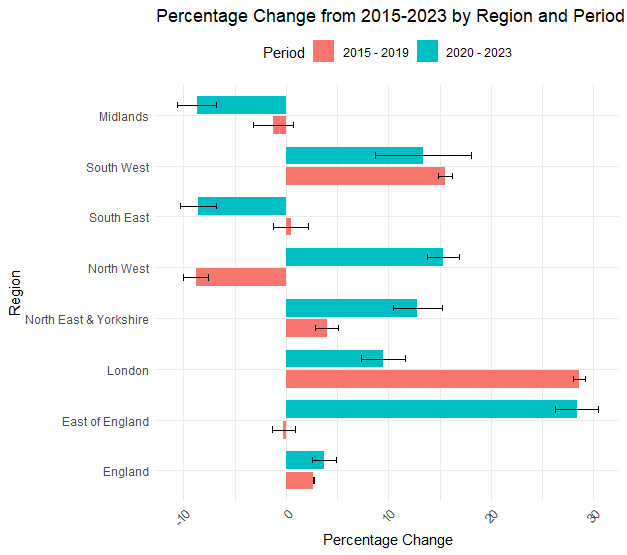


**Figure S1: Mean annual change in deaths per million antipsychotic prescriptions by region and period**
