# Supplementary material for: Trends in recorded deaths involving antipsychotics: The role of deprivation, ethnicity, and regional disparities
Source: PLoS One. 2026 Jun 12;21(6):e0349877. doi: 10.1371/journal.pone.0349877 (PMC13262819; doi:10.1371/journal.pone.0349877)
Supplement: Attachment — Submitted filename: Response to Reviewers Comments.docx [file pone.0349877.s010.docx]

**Reviewer 1**

| **Reviewer’s comment** | **Authors’ response** |
| --- | --- |
| A. Introduction  1. In the first paragraph, the authors correctly note the established efficacy of antipsychotics in severe mental illness. They cite one study in schizophrenia. I suggest adding more studies on the efficacy of antipsychotics in severe mental illness to strengthen the introduction.  10.1016/S0140-6736(19)31135-3 10.1016/j.eclinm.2022.101690 10.1097/MD.0000000000034670 | We thank the reviewer for sharing these studies.  We have now cited these studies in the I*ntroduction (first paragraph).* |
| 2. In addition to established efficacy in treating symptoms, antipsychotics have been associated with reduced mortality in schizophrenia in some population-based studies, for example: 10.1016/j.euroneuro.2025.112745 10.1016/S2215-0366(25)00268-8 This is important to acknowledge in the introduction, and to address in the discussion, especially given the side effect burden and the increase in mortality suggested by this manuscript and other work. | We thank the reviewer for their comment.  We have now cited the suggested studies and have presented a more balanced view of this discussion. The changes are made to the In*troduction (second paragraph)* as well as the *discussion (second paragraph)*. |
| 3. I suggest the authors paraphrase the sentence: “Adverse outcomes associated with antipsychotics include extrapyramidal symptoms (first-generation antipsychotics), metabolic complications (second-generation antipsychotics) and severe events such as poisoning and death” to: “Adverse outcomes associated with antipsychotics include drug-induced movement disorders, metabolic dysfunction, and severe events such as toxicity and death.” I make this suggestion because movement disorders still occur with second-generation agents, and metabolic dysfunction is not exclusive to a single class. | We thank the reviewer for their suggestion.  We have now paraphrased the sentence in line with the reviewer’s suggestion *(Introduction – first paragraph)*. |
| B. Methods 1. The authors need to justify why Asian ethnicity was used as a predictor in the data analysis plan (including prior literature or preliminary analysis). | We have provided further details on why the Asian population was used as a predictor in the methods *(Data extraction and processing – second paragraph).* |
| 2. The authors need to provide more details on what they mean by “an antipsychotic was mentioned on the death certificate.” The names of agents considered antipsychotics need to be listed (perhaps in the supplementary), how this was extracted needs to be described, and whether this can be replicated needs to be clear. The justification for using this pragmatic approach to define “antipsychotic-associated mortality” should be explicitly stated. If this definition cannot be defended as antipsychotic-associated mortality, then the outcome should be renamed to be more accurate. My suggestion is “mortality in people prescribed antipsychotics,” if that is what the data represents. | We thank the reviewer for this important comment.  We agree that greater clarity is needed regarding the definition of “antipsychotic-associated mortality,” and we have now provided the definition in the methods *(Operational definition)*. We have also avoided using the term ‘antipsychotic-associated mortality’ in the manuscript to avoid any confusion and have now used ‘deaths involving antipsychotics’, which more accurately reflects the ONS mortality data. To further enhance transparency and reproducibility, we have provided an additional table in the supplementary information (Table S2) that provides further information on the ICD-codes used by the ONS, providing a clear framework on how deaths were classified as involving antipsychotics based on their mention on death certificates.  Mortality data were extracted from the ONS records of deaths related to drug poisoning in England and Wales, which provides publicly accessible aggregated mortality data. Specifically, we extracted annual counts of deaths involving antipsychotic drugs as reported in the ONS tables. This information has now been provided in the methods. *(Data extraction and processing – first paragraph)*.  As these data are publicly available, the analysis can be replicated by accessing the same ONS datasets and extracting the corresponding yearly counts using the definitions and drug groupings provided.  As suggested by the reviewer, we have provided a list of antipsychotics in the supplementary file (Table S3). |
| 3. Related to the previous point, the methods mention "antipsychotic poisoning" elsewhere which is a completely different clinical diagnosis and this needs to be clarified. | We thank the reviewer for highlighting this important point.  We agree that the term “antipsychotic poisoning” could be potentially misleading, as it implies a specific toxicological diagnosis attributable solely to antipsychotic drugs. We have therefore revised the manuscript to remove the term “antipsychotic poisoning” and replaced it with “deaths involving antipsychotics” throughout, to better reflect the underlying data structure and avoid any implication of causality. |
| C. Results 1. The authors report: “The linear regression analysis at the national level showed a statistically significant positive coefficient, indicating an increasing upward trend (p < 0.01) (Table S2 and Figure 1).” Given that mortality is defined as death certificates in which an antipsychotic is mentioned, the authors also need to report whether prescription counts increased during these years. | We thank the reviewer for this important comment.  In response, we have now provided data on total antipsychotic prescribing counts in England over the study period (2015–2023), which has been included in the Supplementary Material (Table S4). This provides context for the observed trends in deaths involving antipsychotics alongside changes in prescribing volume over time. We have also clarified in the manuscript that mortality rates were calculated per million antipsychotic prescription items to account for changes in prescribing volume. |
| 2. The regional analysis showed significant disparities. These results indicate decreasing rates over time in the Midlands. This suggests that factors other than prescriptions may be driving the rising association with mortality at the national level. It also means the abstract needs revision. In the abstract, the authors state: “the lowest in the Midlands,” but do not state clearly that this is a decrease. I suggest revising the abstract to explicitly state a significant decrease in the Midlands. | We thank the reviewer for this helpful comment.  We agree that the wording in the abstract did not clearly state the direction of change for the Midlands region. We have therefore revised the abstract to explicitly report a statistically significant decrease in the Midlands, consistent with the results presented in the main text *(Abstract – Results)* |
| 3. The authors report models including deprivation and Asian ethnicity, but do not include other major determinants of mortality such as age and sex. Given that these factors could be major contributors to mortality in people prescribed antipsychotics, and not the prescription itself, this limits the interpretability of the results. | We thank the reviewer for this important point.  We agree that age and sex are major determinants of mortality and would ideally be included in the analysis. However, due to the ecological nature of the study and the use of aggregated NHS prescribing data (reported as prescription items rather than individual-level prescriptions), it is not possible to link prescribing exposure to mortality outcomes at an individual level or to stratify prescribing by age and sex.  We have now added this as a limitation in the *Strengths and limitations – (Second paragraph)*, and we have clarified that the findings represent population-level associations rather than individual-level risk estimates. |
| 4. The authors briefly mention that the association between mortality rates and deprivation was non-linear, but do not explain that this could reflect other unexplored variables. | We thank the reviewer for this insightful comment.  We agree that the observed non-linear association between deprivation and mortality rates may reflect the influence of additional unmeasured factors that vary across levels of deprivation, such as healthcare access, comorbidity burden, substance use prevalence, and regional service provision. Therefore, we have revised the manuscript *(Results – last paragraph)* to clarify this point and to emphasise that the non-linear relationship likely reflects the complexity of underlying social and healthcare-related determinants rather than a direct causal effect of deprivation alone. |
| 5. Figure labels need revision. For example, “Linear analysis of Asian and deprivation with antipsychotic death rates” is unclear as Asian is not a directly quantifiable variable. If the model uses proportion of Asian residents, this should be stated. | We thank the reviewer for highlighting this issue.  We agree that the original figure label was unclear and potentially misleading. We have now revised the figure labels to improve clarity and ensure methodological accuracy. The revised figure label now reads: *“Linear analysis of Asian ethnic population proportion, deprivation, and rates of deaths involving antipsychotics.”* |
| D. Discussion and conclusions 1. The authors might want to discuss in detail the possibility that increased mortality associated with antipsychotics may relate, in addition to systemic factors, to overprescription or inappropriate use in populations for whom efficacy and safety has not been established. | We thank the reviewer for this important suggestion.  We agree that the Discussion should acknowledge the possibility that observed mortality patterns may, in part, reflect variation in prescribing appropriateness, including potential overuse or use in populations where the benefit–risk profile of antipsychotics is less well established.  We have now revised the *Discussion (second paragraph)* to include this consideration, with specific reference to older adults and individuals with dementia, where antipsychotics are more frequently prescribed off-label and where evidence regarding long-term safety and efficacy is more limited. |
| 2. One strength of the approach is using deaths per million prescriptions, which partially accounts for increases in prescriptions over time. This should be stated explicitly in the discussion as a strength, while still acknowledging the limitations of the mortality definition. | We thank the reviewer for this helpful suggestion.  We agree that a key strength of the study is the standardisation of mortality rates by prescribing volume, expressed as deaths per million antipsychotic prescription items. We have now explicitly stated this as a strength in the *Strengths and limitations section (First paragraph)*. |
| 3. The analysis of trends before and after COVID-19 is a main strength. | We thank the reviewer for this helpful suggestion.  We agree that the inclusion of a long study period spanning both before and after COVID-19 is a key strength of the study, as it allows for the assessment of temporal changes in deaths involving antipsychotics in the context of a major public health disruption. We have now explicitly highlighted this as a strength in the *Strengths and limitations section (First paragraph).* |
| 4. Including all deaths where an antipsychotic is mentioned on the death certificate creates a major limitation and risks overestimating harm. This needs to be clearly stated in the limitations. | We thank the reviewer for this important comment.  We agree that including all deaths in which an antipsychotic is mentioned on the death certificate may overestimate the extent to which antipsychotics directly contribute to mortality, as this does not establish causality. We have now explicitly acknowledged this limitation in the manuscript *(Strengths and limitations – third paragraph).* |
| 5. Another important point is reverse causation. It is possible that antipsychotics are increasingly prescribed in sicker populations with higher baseline mortality, which would increase the association between antipsychotics and mortality. For example, it is possible that clinicians are treating patients with schizophrenia more frequently with antipsychotics, and schizophrenia is associated with increased mortality, and therefore the trend shows increasing mortality in people prescribed antipsychotics. This possibility needs to be clearly stated. | We thank the reviewer for this important point.  We agree that reverse causation and confounding by indication may contribute to the observed associations, whereby antipsychotics are more likely to be prescribed to individuals with more severe underlying psychiatric illness and higher baseline mortality risk (e.g., schizophrenia or other severe mental disorders). This could partially explain the observed temporal trends in deaths involving antipsychotics, independent of any direct effect of the medications themselves. We have now explicitly included this limitation in the *Strengths and limitations section (Fourth paragraph).* |
| 6. I believe the conclusions are overstated given the outcome definition and the lack of key mortality determinants in the models and also given that regional findings include a significant decrease in the Midlands. The possibility of reverse causation and overestimation of mortality are major limitations. | We thank the reviewer for this important comment, and we agree with it.  We have revised the Conclusions to use more cautious language, avoiding causal interpretations and to better reflect the ecological nature of the analysis. We have also explicitly acknowledged regional heterogeneity, including the observed decrease in the Midlands, and reframed the findings as population-level associations. |

**Reviewer 2**

| **Reviewer’s Comment** | **Authors’ Response** |
| --- | --- |
| 1. The authors define mortality based on the mention of an antipsychotic on the death certificate, but it remains unclear whether these deaths were primarily due to acute toxicity such as poisoning or chronic physical complications like cardiovascular events associated with long-term use; therefore, please clarify if the ONS data used distinguish between "deaths due to drug poisoning" and "deaths where antipsychotics were a contributing factor," and discuss how this distinction relates to the overall increased mortality trends described in the study. | We thank the reviewer for this important clarification.  The ONS dataset used in this study is restricted to drug poisoning deaths, as defined by ICD-10 codes, in which substances mentioned on the death certificate are recorded. It does not include deaths due to chronic physical complications associated with long-term antipsychotic use. We have now clarified this distinction in the *Methods (Operational definition) and Discussion sections (First paragraph).* |
| 2. While the study uses prescription item counts as a denominator to evaluate mortality rates, regional variations in these outcomes might reflect differences in the underlying prevalence and severity of mental illnesses rather than the pharmacological effects of the medications themselves; consequently, the authors should discuss how regional disparities in the prevalence of severe mental illness might confound the observed association between deprivation and mortality. | We thank the reviewer for this important point.  We agree that regional variation in the prevalence and severity of severe mental illness may contribute to the observed differences in mortality rates and could confound associations with deprivation. We have now included this consideration in the *Discussion (third paragraph) and Strengths and limitations section (fourth paragraph).* |
| 3. To move beyond simple association and better address potential causality, it is necessary for the authors to provide more granular data—if available—regarding the specific causes of death associated with different classes of antipsychotics (e.g., first-generation vs. second-generation); this would allow for a more robust discussion on whether the increased mortality is driven by acute pharmacological toxicity, metabolic complications, or systemic inequalities in the monitoring of high-risk patients. | We thank the reviewer for this important suggestion.  We agree that more granular analyses examining cause-specific mortality and differences between antipsychotic classes (e.g., first- versus second-generation agents) would provide valuable insights into potential mechanisms underlying the observed trends. However, the ONS dataset used in this study is limited to aggregated data and does not allow for detailed stratification by specific causes of death or by antipsychotic class in a way that would support causal inference.  We have now clarified this issue in the manuscript and highlighted the need for future research using individual-level, linked datasets to explore cause-specific mortality and differential risks across antipsychotic classes *(Discussion – first paragraph; Strengths and limitations – fourth paragraph).* |

**Reviewer 3**

| **Reviewer’s Comment** | **Authors’ Response** |
| --- | --- |
| Introduction: Recommend removing the specifiers of first generation antipsychotics with extrapyramidal symptoms and second generation antipsychotics with metabolic complications as agents in both classes can cause both types of adverse effects (even if they are more prominent in one class vs. the other) | We thank the reviewer for their suggestion.  We have now paraphrased the sentence in line with the reviewer’s suggestion *(Introduction – first paragraph)*. |
| You correctly state that there is inconclusive evidence on the effect of antipsychotics on mortality, but the objective in your abstract clearly states that antipsychotics are associated with increased mortality. This must be rectified. | We thank the reviewer for highlighting this inconsistency.  We agree and have revised the *abstract (objective)* to reflect the uncertainty in the relationship between antipsychotic use and mortality, ensuring consistency with the Introduction and current evidence base. |
| Please define “deprivation”- as a reader from outside of the UK I am not familiar with this term being used in the context of socioeconomic disadvantage and it was not clear to me whether you were referring to individuals lacking a specific resource. | We thank the reviewer for this helpful comment.  We have now clarified in the *Methods section (Data design and sources)* that deprivation refers to a composite area-level measure of socioeconomic disadvantage, as captured by the English Indices of Deprivation. |
| Please elaborate on the idea of ethnicity being protective against psychoses- how would this influence antipsychotic-related mortality? | We thank the reviewer for this important comment.  We have clarified that our discussion refers to ethnic density rather than ethnicity itself and have expanded the text to explain how its potential protective effects on psychosis incidence may indirectly influence antipsychotic prescribing patterns, service engagement, and consequently deaths involving antipsychotics *(Introduction – fourth paragraph).* |
| Please elaborate on how prescribing patterns influence mortality and how understanding disparities can influence prescribing patterns to improve outcomes. If the links between these concepts were more clearly laid out and/or the scope was more narrowly defined, that could strengthen the generalizability of your study. | We thank the reviewer for this helpful comment.  We have expanded the *Introduction (third paragraph) and Discussion (third paragraph)* to clarify how variations in prescribing patterns may influence mortality risk and how understanding regional and population-level differences in prescribing may help identify inequalities in care and inform safer and more equitable prescribing practices. |
| Methods: Can you do an analysis comparing the change in mortality rate over time stratified by the different regions/ethnic/socioeconomic groups? I am curious if the overall trend is driven by any particular subgroup | We thank the reviewer for this insightful suggestion.  We agree that examining temporal trends stratified by region, deprivation, and ethnic composition would further enhance understanding of subgroup-specific drivers of the overall trend. In the current study, formal temporal trend analyses were only feasible at the regional level, as shown in Figure 1, as annual regional mortality rates were available, allowing assessment of changes over time within regions. In contrast, deprivation and ethnic density were derived from area-level socioeconomic and demographic characteristics that are relatively stable over time at the regional scale and therefore were analysed as contextual variables rather than time-varying exposures. As a result, formal modelling of temporal changes across these strata was not possible within the available data structure. We have clarified this limitation in the manuscript and highlighted this as an important area for future research using more granular, longitudinal datasets *(Strengths and limitations – last paragraph)*. |
| Discussion: You state that “a cautious and personalised approach to treatment should be encouraged, considering the adverse effects and variation in prescribing and cultural beliefs and practices.” Can you provide a specific example to better illustrate this point? Or reference literature that describes this type of approach in a tangible way that clinicians reading your paper may be able to incorporate into their practice? | We thank the reviewer for this helpful suggestion.  We have now supplemented this statement with relevant literature describing personalised prescribing approaches that incorporate individual risk profiles and adverse effect monitoring to guide safer antipsychotic use in clinical practice *(Discussion – sixth paragraph).* |
| The claim that your study “emphasises the importance of personalised treatment in managing patients on antipsychotic medications” may be a bit of a leap for a population-level analysis, which as you mentioned in the limitations does not include individual patient data or control for confounding. | We thank the reviewer for this comment.  We agree that the original wording may overstate the clinical implications of our population-level findings. We have therefore removed the statement referring to personalised treatment in the section Implications for practice, policy and research. |
| Figures 3 and 4: Please clarify the axis labels for these graphs with units. | Thank you for this comment.  We have revised both figures to clarify the axis labels and improve interpretability. For all figures, the predictor axes now explicitly state the measurement scales (e.g. ethnic density expressed as a percentage of the population and deprivation measured using the Index of Multiple Deprivation score). For figures presenting GAM smooths, the y‑axis has been clarified to indicate that it represents model‑estimated partial (smooth) effects on the outcome, which are centred at zero and do not correspond to observed mortality units. Corresponding clarifications have been added to the figures. |
| Overall: The abstract and introduction must be fair and balanced when setting the stage for an investigation on the effects of antipsychotic medications on mortality. There is evidence for these agents both increasing and decreasing mortality, so both must be presented in an objective way. | We thank the reviewer for this important comment.  We have revised both the *Abstract (Objective)* and *Introduction (second paragraph)* to provide a more balanced and objective overview of the literature, explicitly acknowledging that existing evidence suggests both potential increases and decreases in mortality associated with antipsychotic use. |
| I think it is important to note that your study only included outpatients, which tend to be a less severe population. This should be discussed in terms of the effect on generalizability of your findings. | We thank the reviewer for this comment.  We acknowledge that the prescription data primarily reflect community-dispensed medications, which may underrepresent antipsychotic use in inpatient settings or more severe cases managed in secondary care. We have clarified this in the manuscript *(Strengths and limitations – fifth paragraph).* |
| I came away unsure what you hypothesized was causing antipsychotic-related mortality to increase over time. You suggest the use of high doses in older patients but don’t share any evidence to support this practice is becoming more common. It seems like the opposite trend would be expected since our knowledge of monitoring for and identifying adverse effects has improved over time and the agents that have come on the market in the last 10 years have all generally had more favorable metabolic profiles and/or been LAIs. | We thank the reviewer for this important comment.  We would like to clarify that the primary aim of this study was descriptive, to examine temporal trends in deaths involving antipsychotics and their variation across regions and population groups, rather than to test specific causal hypotheses regarding the underlying drivers of these trends. However, we agree that the original text may have implied potential explanations, such as dosing or treatment phase, without sufficient evidence to support their role in explaining temporal changes.  We have therefore revised the Discussion to clarify the descriptive nature of the study and to avoid speculative interpretations *(Discussion – first paragraph)*. In particular, we have acknowledged that factors such as dosing were not directly examined in this study *(Discussion – second paragraph)*. We have also modified the language to present these factors as potential mechanisms reported in the literature rather than as drivers of the findings. In addition, we have acknowledged that observed trends may reflect multiple contributing factors, such as including changes in prescribing practices and patient characteristics *(Discussion – second paragraph)*.  Furthermore, we agree that prescribing practices in older adults have become more cautious over time, with increased awareness of adverse effects, improved monitoring, and greater use of agents with more favourable metabolic profiles and long-acting formulations. Therefore, we have clarified that older adults represent a clinically vulnerable population with increased susceptibility to adverse effects even at standard doses, and that observed mortality patterns likely reflect a combination of dose-related effects, treatment initiation, and patient-level vulnerability rather than prescribing intensity alone *(Discussion – second paragraph)*. |
| Your conclusions focus on the importance of considering patient characteristics such as geographic region, socioeconomic status, and ethnicity rather than exploring the reasons behind the observed increase in antipsychotic-related mortality over time. I believe a meaningful way to tie these concepts together is to analyze the effects of region/deprivation/ethnicity on the rate of change in antipsychotic-related mortality over time to determine the relative impact of each of these variables. It seems like the ultimate goal should be to reduce the mortality rate associated with antipsychotics and if we had some clues as to what is driving the rate increase it would helpful for developing mitigation strategies. Deprescribing and the use of non-pharmacological therapy aren’t strategies to reduce the rate of antipsychotic-related mortality per prescription. | We thank the reviewer for this insightful suggestion.  We agree that examining whether the observed increase in antipsychotic-related mortality is driven by specific population subgroups over time would provide important additional insight into potential underlying mechanisms.  In the present study, we were able to assess temporal trends at the regional level using annual mortality rates, as shown in Figure 1. However, deprivation and ethnic composition were derived from area-level census-based indicators that are relatively stable over the study period at the regional scale. As such, these variables were treated as contextual characteristics rather than time-varying exposures, and formal modelling of changes in mortality trends across these strata (i.e., interaction with time) was not methodologically feasible within the available data structure. We have clarified this limitation in the manuscript *(Strengths and limitations – last paragraph)*.  We also agree that future studies using more granular longitudinal or individual-level data would be better suited to formally assess whether temporal increases in mortality are differentially driven by specific regional, socioeconomic, or ethnic subgroups, and to identify the underlying mechanisms contributing to these patterns. Therefore, we have added this suggestion to the *Conclusions.* |
| You conclude that regional differences in prescribing patterns contribute to disparities, but at the population level it is impossible to attribute the observed regional differences to prescribing patterns specifically. You also mention access to resources as a regional variable. It would be more accurate to state that you observed regional difference and not speculate at the driving force. | We thank the reviewer for this helpful comment.  We agree that attributing regional differences to specific drivers is not supported by the ecological nature of the data. We have therefore *revised the Conclusions* to remove causal interpretations and now present these findings in more neutral, descriptive terms focused on observed regional heterogeneity. |
