# Supplementary material for: Trends in recorded deaths involving antipsychotics: The role of deprivation, ethnicity, and regional disparities
Source: PLoS One. 2026 Jun 12;21(6):e0349877. doi: 10.1371/journal.pone.0349877 (PMC13262819; doi:10.1371/journal.pone.0349877)
Supplement: Attachment — Submitted filename: Response_to_Reviewers_Comments_auresp_2.docx [file pone.0349877.s011.docx]

**Reviewer 1**

| **Reviewer’s comment** | **Authors’ response** |
| --- | --- |
| I would like to thank the authors for addressing my comments.  The manuscript has improved significantly, and now clarifies the outcome "Deaths involving Antipsychotics" and specifies how that outcome was operationalized. | We thank the reviewer for their positive feedback and are pleased that the revisions have addressed the concerns raised. |

**Reviewer 2**

| **Reviewer’s Comment** | **Authors’ Response** |
| --- | --- |
| Reviewer #2: As the authors adequately revised the manuscript according to the reviewers' comments, I have no further comments and think that the current version is now acceptable. | We thank the reviewer for their positive feedback and are pleased that the revisions have addressed all concerns. |

**Reviewer 3**

| **Reviewer’s Comment** | **Authors’ Response** |
| --- | --- |
| 1) I still think more could be done to establish the link between ethnic density and antipsychotic mortality. You cited literature about ethnic density protecting against psychosis but not how this translates to reduced antipsychotic-related mortality. Can you provide any evidence to support better cardiovascular and/or metabolic health in these areas that could counteract the adverse events seen with antipsychotics? If you are attributing the protective effect to "better engagement with healthcare services" then please expand on how specifically this could reduce antipsychotic mortality. You do not have to state a claim, just hypothesize as to what might be driving your finding. | We thank the reviewer for this insightful comment.  We have expanded the Discussion (7^th^ Paragraph) to further elaborate on potential mechanisms linking ethnic density and deaths involving antipsychotics. Specifically, we have clarified how higher ethnic density may be associated with improved healthcare engagement, which could facilitate closer monitoring for adverse effects, more appropriate prescribing, and improved adherence, thereby potentially influencing outcomes. These explanations are presented as hypotheses rather than causal interpretations. |
| 2) You do a nice job discussing potential mechanisms underlying antipsychotic mortality, but I am still left to wonder why it is increasing over time. You brought up dosing- do you have evidence that dosing is increasing over time? Does this in any way explain the regional differences you observed? Same a above, you do not have to state a claim but hypothesize as to what might underly your main finding. | We thank the reviewer for this important comment.  We agree that the observed increase in mortality over time warrants further interpretation. We have expanded the Discussion (2^nd^ paragraph) to clarify that the increasing trend may plausibly reflect a combination of factors. These include potential changes in prescribing practices over time, such as greater use of antipsychotic polypharmacy, alongside differences in service capacity and monitoring intensity across regions. We have also emphasised that these explanations are speculative and intended to generate hypotheses for future research rather than confirm causal mechanisms. |
