# Supplementary material for: Trends in recorded deaths involving antipsychotics: The role of deprivation, ethnicity, and regional disparities
Source: PLoS One. 2026 Jun 12;21(6):e0349877. doi: 10.1371/journal.pone.0349877 (PMC13262819; doi:10.1371/journal.pone.0349877)
Supplement: Peer review history

# Peer review: Decision Letter 0

**DOI:** 10.1371/journal.pone.0349877.r001

**Authors:** Hikaru Hori

7 Mar 2026

PONE-D-25-54201

Rising Trends in Antipsychotic-Associated Mortality: The Role of Deprivation, Ethnicity, and Regional Disparities

PLOS One

Dear Dr. Khan,

Thank you for submitting your manuscript to PLOS ONE. After careful consideration, we feel that it has merit but does not fully meet PLOS ONE’s publication criteria as it currently stands. Therefore, we invite you to submit a revised version of the manuscript that addresses the points raised during the review process.

Please submit your revised manuscript by Apr 21 2026 11:59PM. If you will need more time than this to complete your revisions, please reply to this message or contact the journal office at plosone@plos.org. When you're ready to submit your revision, log on to https://www.editorialmanager.com/pone/ and select the 'Submissions Needing Revision' folder to locate your manuscript file.

Please include the following items when submitting your revised manuscript:

- A letter that responds to each point raised by the academic editor and reviewer(s). You should upload this letter as a separate file labeled 'Response to Reviewers'.
- A marked-up copy of your manuscript that highlights changes made to the original version. You should upload this as a separate file labeled 'Revised Manuscript with Track Changes'.
- An unmarked version of your revised paper without tracked changes. You should upload this as a separate file labeled 'Manuscript'.

If you would like to make changes to your financial disclosure, please include your updated statement in your cover letter. Guidelines for resubmitting your figure files are available below the reviewer comments at the end of this letter.

If applicable, we recommend that you deposit your laboratory protocols in protocols.io to enhance the reproducibility of your results. Protocols.io assigns your protocol its own identifier (DOI) so that it can be cited independently in the future. For instructions see: https://journals.plos.org/plosone/s/submission-guidelines#loc-laboratory-protocols. Additionally, PLOS ONE offers an option for publishing peer-reviewed Lab Protocol articles, which describe protocols hosted on protocols.io. Read more information on sharing protocols at https://plos.org/protocols?utm_medium=editorial-email&utm_source=authorletters&utm_campaign=protocols.

We look forward to receiving your revised manuscript.

Kind regards,

Hikaru Hori

Academic Editor

PLOS One

Journal Requirements:

When submitting your revision, we need you to address these additional requirements.

1. Please ensure that your manuscript meets PLOS ONE's style requirements, including those for file naming. The PLOS ONE style templates can be found at

https://journals.plos.org/plosone/s/file?id=wjVg/PLOSOne_formatting_sample_main_body.pdf and

https://journals.plos.org/plosone/s/file?id=ba62/PLOSOne_formatting_sample_title_authors_affiliations.pdf

2. If the reviewer comments include a recommendation to cite specific previously published works, please review and evaluate these publications to determine whether they are relevant and should be cited. There is no requirement to cite these works unless the editor has indicated otherwise.

[Note: HTML markup is below. Please do not edit.]

Reviewers' comments:

Reviewer's Responses to Questions

**Comments to the Author**

1. Is the manuscript technically sound, and do the data support the conclusions?

The manuscript must describe a technically sound piece of scientific research with data that supports the conclusions. Experiments must have been conducted rigorously, with appropriate controls, replication, and sample sizes. The conclusions must be drawn appropriately based on the data presented.

Reviewer #1: No

Reviewer #2: Partly

Reviewer #3: Partly

**********

2. Has the statistical analysis been performed appropriately and rigorously?

Reviewer #1: No

Reviewer #2: No

Reviewer #3: I Don't Know

**********

3. Have the authors made all data underlying the findings in their manuscript fully available?

The PLOS Data policy requires authors to make all data underlying the findings described in their manuscript fully available without restriction, with rare exception (please refer to the Data Availability Statement in the manuscript PDF file). The data should be provided as part of the manuscript or its supporting information, or deposited to a public repository. For example, in addition to summary statistics, the data points behind means, medians and variance measures should be available. If there are restrictions on publicly sharing data—e.g. participant privacy or use of data from a third party—those must be specified.

Reviewer #1: Yes

Reviewer #2: No

Reviewer #3: Yes

**********

4. Is the manuscript presented in an intelligible fashion and written in standard English?

PLOS ONE does not copyedit accepted manuscripts, so the language in submitted articles must be clear, correct, and unambiguous. Any typographical or grammatical errors should be corrected at revision, so please note any specific errors here.

Reviewer #1: Yes

Reviewer #2: Yes

Reviewer #3: Yes

**********

5. Review Comments to the Author

Please use the space provided to explain your answers to the questions above. You may also include additional comments for the author, including concerns about dual publication, research ethics, or publication ethics. (Please upload your review as an attachment if it exceeds 20,000 characters)

Reviewer #1: Jan 21, 2026

Peer review report

Dear Editor,

Thank you for the opportunity to review this manuscript titled “Rising Trends in Antipsychotic-Associated Mortality: The Role of Deprivation, Ethnicity, and Regional Disparities.”

I first present my summary of what I understood after carefully reading through the manuscript, then I provide my detailed comments.

Summary:

This is a population-based analysis of national data in England. Prescription data were obtained from the NHS Business Services Authority (NHSBSA), and mortality data were obtained from the UK Office for National Statistics (ONS). The authors examine the association between antipsychotic prescriptions and “antipsychotic-related mortality.” Unfortunately, the authors define antipsychotic-related mortality as “records of deaths where an antipsychotic was mentioned on the death certificate,” and it is unclear what this represents. It is not clear whether this reflects antipsychotic poisoning (as the authors mention elsewhere) or mortality in people prescribed antipsychotics, which is not the same as antipsychotic-associated mortality and would undermine the results of this analysis.

The authors then analyze the relationship between mortality and antipsychotic prescriptions across NHS England regions, as well as ethnic and deprivation factors (income, employment, education, and health). The authors analyze data from 2015 to 2023 and conclude that average antipsychotic-related mortality per million prescriptions has increased over the past decade, with the increase mostly in more urbanized regions (for example London). The Midlands, however, show a significantly decreasing rate, and the abstract does not report this accurately. In addition, the authors do not report the change in prescribing patterns which is important in interpreting the results.

The main issue I have with this work is the definition of antipsychotic-associated mortality as any death certificate where an antipsychotic is mentioned. This pragmatic definition is not defended, not clearly detailed, and risks overestimating antipsychotic-associated mortality. It also makes it difficult to interpret regional and deprivation effects as “antipsychotic-associated mortality.” Another major issue is the lack of other variables that can explain mortality patterns, such as age, sex, and chronic medical illness.

I believe the title, abstract, and main text need to be revised to clearly convey what the authors actually analyzed. If the outcome is closer to “mortality in people prescribed antipsychotics,” this should be stated explicitly and consistently. If the outcome is antipsychotic poisoning, then the methods and outcome definition need to match this claim. In addition, key sociodemographic variables that determine mortality need to be included to clarify the relationship between prescriptions and mortality. The discussion should be expanded to fully explore strengths and limitations.

Detailed comments:

A. Introduction

1. In the first paragraph, the authors correctly note the established efficacy of antipsychotics in severe mental illness. They cite one study in schizophrenia. I suggest adding more studies on the efficacy of antipsychotics in severe mental illness to strengthen the introduction:

10.1016/S0140-6736(19)31135-3

10.1016/j.eclinm.2022.101690

10.1097/MD.0000000000034670

2. In addition to established efficacy in treating symptoms, antipsychotics have been associated with reduced mortality in schizophrenia in some population-based studies, for example:

10.1016/j.euroneuro.2025.112745

10.1016/S2215-0366(25)00268-8

This is important to acknowledge in the introduction, and to address in the discussion, especially given the side effect burden and the increase in mortality suggested by this manuscript and other work.

3. I suggest the authors paraphrase the sentence: “Adverse outcomes associated with antipsychotics include extrapyramidal symptoms (first-generation antipsychotics), metabolic complications (second-generation antipsychotics) and severe events such as poisoning and death” to: “Adverse outcomes associated with antipsychotics include drug-induced movement disorders, metabolic dysfunction, and severe events such as toxicity and death.”

I make this suggestion because movement disorders still occur with second-generation agents, and metabolic dysfunction is not exclusive to a single class.

B. Methods

1. The authors need to justify why Asian ethnicity was used as a predictor in the data analysis plan (including prior literature or preliminary analysis).

2. The authors need to provide more details on what they mean by “an antipsychotic was mentioned on the death certificate.” The names of agents considered antipsychotics need to be listed (perhaps in the supplementary), how this was extracted needs to be described, and whether this can be replicated needs to be clear. The justification for using this pragmatic approach to define “antipsychotic-associated mortality” should be explicitly stated. If this definition cannot be defended as antipsychotic-associated mortality, then the outcome should be renamed to be more accurate. My suggestion is “mortality in people prescribed antipsychotics,” if that is what the data represents.

3. Related to the previous point, the methods mention "antipsychotic poisoning" elsewhere which is a completely different clinical diagnosis and this needs to be clarified.

C. Results

1. The authors report:

“The linear regression analysis at the national level showed a statistically significant positive coefficient, indicating an increasing upward trend (p < 0.01) (Table S2 and Figure 1).” Given that mortality is defined as death certificates in which an antipsychotic is mentioned, the authors also need to report whether prescription counts increased during these years.

2. The regional analysis showed significant disparities. These results indicate decreasing rates over time in the Midlands. This suggests that factors other than prescriptions may be driving the rising association with mortality at the national level. It also means the abstract needs revision. In the abstract, the authors state: “the lowest in the Midlands,” but do not state clearly that this is a decrease. I suggest revising the abstract to explicitly state a significant decrease in the Midlands.

3. The authors report models including deprivation and Asian ethnicity, but do not include other major determinants of mortality such as age and sex. Given that these factors could be major contributors to mortality in people prescribed antipsychotics, and not the prescription itself, this limits the interpretability of the results.

4. The authors briefly mention that the association between mortality rates and deprivation was non-linear, but do not explain that this could reflect other unexplored variables.

5. Figure labels need revision. For example, “Linear analysis of Asian and deprivation with antipsychotic death rates” is unclear as Asian is not a directly quantifiable variable. If the model uses proportion of Asian residents, this should be stated.

D. Discussion and conclusions

1. The authors might want to discuss in detail the possibility that increased mortality associated with antipsychotics may relate, in addition to systemic factors, to overprescription or inappropriate use in populations for whom efficacy and safety has not been established.

2. One strength of the approach is using deaths per million prescriptions, which partially accounts for increases in prescriptions over time. This should be stated explicitly in the discussion as a strength, while still acknowledging the limitations of the mortality definition.

3. The analysis of trends before and after COVID-19 is a main strength.

4. Including all deaths where an antipsychotic is mentioned on the death certificate creates a major limitation and risks overestimating harm. This needs to be clearly stated in the limitations.

5. Another important point is reverse causation. It is possible that antipsychotics are increasingly prescribed in sicker populations with higher baseline mortality, which would increase the association between antipsychotics and mortality. For example, it is possible that clinicians are treating patients with schizophrenia more frequently with antipsychotics, and schizophrenia is associated with increased mortality, and therefore the trend shows increasing mortality in people prescribed antipsychotics. This possibility needs to be clearly stated.

6. I believe the conclusions are overstated given the outcome definition and the lack of key mortality determinants in the models and also given that regional findings include a significant decrease in the Midlands. The possibility of reverse causation and overestimation of mortality are major limitations.

Thank you once again for the opportunity to review this manuscript.

Reviewer #2: This study addresses an important public health issue by analyzing national-level data on antipsychotic-associated mortality in England over nearly a decade. The findings regarding the rising mortality rate and its association with regional deprivation and ethnic density are compelling and suggest a need for targeted clinical and policy interventions. However, the manuscript requires further clarification on its methodology and a more nuanced interpretation of the findings, particularly regarding the potential confounding factors inherent in population-level ecological studies.

1. The authors define mortality based on the mention of an antipsychotic on the death certificate, but it remains unclear whether these deaths were primarily due to acute toxicity such as poisoning or chronic physical complications like cardiovascular events associated with long-term use; therefore, please clarify if the ONS data used distinguish between "deaths due to drug poisoning" and "deaths where antipsychotics were a contributing factor," and discuss how this distinction relates to the overall increased mortality trends described in the study.

2. While the study uses prescription item counts as a denominator to evaluate mortality rates, regional variations in these outcomes might reflect differences in the underlying prevalence and severity of mental illnesses rather than the pharmacological effects of the medications themselves; consequently, the authors should discuss how regional disparities in the prevalence of severe mental illness might confound the observed association between deprivation and mortality.

3. To move beyond simple association and better address potential causality, it is necessary for the authors to provide more granular data—if available—regarding the specific causes of death associated with different classes of antipsychotics (e.g., first-generation vs. second-generation); this would allow for a more robust discussion on whether the increased mortality is driven by acute pharmacological toxicity, metabolic complications, or systemic inequalities in the monitoring of high-risk patients.

Reviewer #3: Introduction:

• Recommend removing the specifiers of first generation antipsychotics with extrapyramidal symptoms and second generation antipsychotics with metabolic complications as agents in both classes can cause both types of adverse effects (even if they are more prominent in one class vs. the other)

• You correctly state that there is inconclusive evidence on the effect of antipsychotics on mortality, but the objective in your abstract clearly states that antipsychotics are associated with increased mortality. This must be rectified.

• Please define “deprivation”- as a reader from outside of the UK I am not familiar with this term being used in the context of socioeconomic disadvantage and it was not clear to me whether you were referring to individuals lacking a specific resource.

• Please elaborate on the idea of ethnicity being protective against psychoses- how would this influence antipsychotic-related mortality?

• Please elaborate on how prescribing patterns influence mortality and how understanding disparities can influence prescribing patterns to improve outcomes. If the links between these concepts were more clearly laid out and/or the scope was more narrowly defined, that could strengthen the generalizability of your study.

Methods:

• Can you do an analysis comparing the change in mortality rate over time stratified by the different regions/ethnic/socioeconomic groups? I am curious if the overall trend is driven by any particular subgroup.

Discussion:

• You state that “a cautious and personalised approach to treatment should be encouraged, considering the adverse effects and variation in prescribing and cultural beliefs and practices.” Can you provide a specific example to better illustrate this point? Or reference literature that describes this type of approach in a tangible way that clinicians reading your paper may be able to incorporate into their practice?

• The claim that your study “emphasises the importance of personalised treatment in managing patients on antipsychotic medications” may be a bit of a leap for a population-level analysis, which as you mentioned in the limitations does not include individual patient data or control for confounding.

Figures 3 and 4:

• Please clarify the axis labels for these graphs with units.

Overall:

• The abstract and introduction must be fair and balanced when setting the stage for an investigation on the effects of antipsychotic medications on mortality. There is evidence for these agents both increasing and decreasing mortality, so both must be presented in an objective way.

• I think it is important to note that your study only included outpatients, which tend to be a less severe population. This should be discussed in terms of the effect on generalizability of your findings.

• I came away unsure what you hypothesized was causing antipsychotic-related mortality to increase over time. You suggest the use of high doses in older patients but don’t share any evidence to support this practice is becoming more common. It seems like the opposite trend would be expected since our knowledge of monitoring for and identifying adverse effects has improved over time and the agents that have come on the market in the last 10 years have all generally had more favorable metabolic profiles and/or been LAIs.

• Your conclusions focus on the importance of considering patient characteristics such as geographic region, socioeconomic status, and ethnicity rather than exploring the reasons behind the observed increase in antipsychotic-related mortality over time. I believe a meaningful way to tie these concepts together is to analyze the effects of region/deprivation/ethnicity on the rate of change in antipsychotic-related mortality over time to determine the relative impact of each of these variables. It seems like the ultimate goal should be to reduce the mortality rate associated with antipsychotics and if we had some clues as to what is driving the rate increase it would helpful for developing mitigation strategies. Deprescribing and the use of non-pharmacological therapy aren’t strategies to reduce the rate of antipsychotic-related mortality per prescription.

• You conclude that regional differences in prescribing patterns contribute to disparities, but at the population level it is impossible to attribute the observed regional differences to prescribing patterns specifically. You also mention access to resources as a regional variable. It would be more accurate to state that you observed regional difference and not speculate at the driving force.

**********

6. PLOS authors have the option to publish the peer review history of their article (what does this mean?). If published, this will include your full peer review and any attached files.

If you choose “no”, your identity will remain anonymous but your review may still be made public.

**Do you want your identity to be public for this peer review?**  For information about this choice, including consent withdrawal, please see our Privacy Policy.

Reviewer #1: No

Reviewer #2: No

Reviewer #3: **Yes:** Shaina Schwartz

**********

[NOTE: If reviewer comments were submitted as an attachment file, they will be attached to this email and accessible via the submission site. Please log into your account, locate the manuscript record, and check for the action link "View Attachments". If this link does not appear, there are no attachment files.]

To ensure your figures meet our technical requirements, please review our figure guidelines: https://journals.plos.org/plosone/s/figures

You may also use PLOS’s free figure tool, NAAS, to help you prepare publication quality figures: https://journals.plos.org/plosone/s/figures#loc-tools-for-figure-preparation.

NAAS will assess whether your figures meet our technical requirements by comparing each figure against our figure specifications.

# Author comment: Author response to Decision Letter 1

**DOI:** 10.1371/journal.pone.0349877.r002

13 Apr 2026

Please see the attached document titled 'Response to Reviewers' Comments.

Attachment Submitted filename: Response to Reviewers Comments.docx

# Peer review: Decision Letter 1

**DOI:** 10.1371/journal.pone.0349877.r003

**Authors:** Hikaru Hori

22 Apr 2026

PONE-D-25-54201R1

Rising Trends in Deaths involving Antipsychotics: The Role of Deprivation, Ethnicity, and Regional Disparities

PLOS One

Dear Dr. Khan,

Thank you for submitting your manuscript to PLOS ONE. After careful consideration, we feel that it has merit but does not fully meet PLOS ONE’s publication criteria as it currently stands. Therefore, we invite you to submit a revised version of the manuscript that addresses the points raised during the review process.

Please submit your revised manuscript by Jun 06 2026 11:59PM. If you will need more time than this to complete your revisions, please reply to this message or contact the journal office at plosone@plos.org. When you're ready to submit your revision, log on to https://www.editorialmanager.com/pone/ and select the 'Submissions Needing Revision' folder to locate your manuscript file.

Please include the following items when submitting your revised manuscript:

- A letter that responds to each point raised by the academic editor and reviewer(s). You should upload this letter as a separate file labeled 'Response to Reviewers'.
- A marked-up copy of your manuscript that highlights changes made to the original version. You should upload this as a separate file labeled 'Revised Manuscript with Track Changes'.
- An unmarked version of your revised paper without tracked changes. You should upload this as a separate file labeled 'Manuscript'.

If you would like to make changes to your financial disclosure, please include your updated statement in your cover letter. Guidelines for resubmitting your figure files are available below the reviewer comments at the end of this letter.

If applicable, we recommend that you deposit your laboratory protocols in protocols.io to enhance the reproducibility of your results. Protocols.io assigns your protocol its own identifier (DOI) so that it can be cited independently in the future. For instructions see: https://journals.plos.org/plosone/s/submission-guidelines#loc-laboratory-protocols. Additionally, PLOS ONE offers an option for publishing peer-reviewed Lab Protocol articles, which describe protocols hosted on protocols.io. Read more information on sharing protocols at https://plos.org/protocols?utm_medium=editorial-email&utm_source=authorletters&utm_campaign=protocols.

As the corresponding author, your ORCID iD is verified in the submission system and will appear in the published article. PLOS supports the use of ORCID, and we encourage all coauthors to register for an ORCID iD and use it as well. Please encourage your coauthors to verify their ORCID iD within the submission system before final acceptance, as unverified ORCID iDs will not appear in the published article. *Only*  the individual author can complete the verification step; PLOS staff *cannot*  verify ORCID iDs on behalf of authors.

We look forward to receiving your revised manuscript.

Kind regards,

Hikaru Hori

Academic Editor

PLOS One

Journal Requirements:

1. If the reviewer comments include a recommendation to cite specific previously published works, please review and evaluate these publications to determine whether they are relevant and should be cited. There is no requirement to cite these works unless the editor has indicated otherwise.

2. Please review your reference list to ensure that it is complete and correct. If you have cited papers that have been retracted, please include the rationale for doing so in the manuscript text, or remove these references and replace them with relevant current references. Any changes to the reference list should be mentioned in the rebuttal letter that accompanies your revised manuscript. If you need to cite a retracted article, indicate the article’s retracted status in the References list and also include a citation and full reference for the retraction notice.

[Note: HTML markup is below. Please do not edit.]

Reviewers' comments:

Reviewer's Responses to Questions

**Comments to the Author**

1. If the authors have adequately addressed your comments raised in a previous round of review and you feel that this manuscript is now acceptable for publication, you may indicate that here to bypass the “Comments to the Author” section, enter your conflict of interest statement in the “Confidential to Editor” section, and submit your "Accept" recommendation.

Reviewer #1: All comments have been addressed

Reviewer #2: All comments have been addressed

Reviewer #3: All comments have been addressed

**********

2. Is the manuscript technically sound, and do the data support the conclusions?

The manuscript must describe a technically sound piece of scientific research with data that supports the conclusions. Experiments must have been conducted rigorously, with appropriate controls, replication, and sample sizes. The conclusions must be drawn appropriately based on the data presented.

Reviewer #1: Yes

Reviewer #2: Yes

Reviewer #3: Yes

**********

3. Has the statistical analysis been performed appropriately and rigorously?

Reviewer #1: Yes

Reviewer #2: Yes

Reviewer #3: I Don't Know

**********

4. Have the authors made all data underlying the findings in their manuscript fully available?

The PLOS Data policy requires authors to make all data underlying the findings described in their manuscript fully available without restriction, with rare exception (please refer to the Data Availability Statement in the manuscript PDF file). The data should be provided as part of the manuscript or its supporting information, or deposited to a public repository. For example, in addition to summary statistics, the data points behind means, medians and variance measures should be available. If there are restrictions on publicly sharing data—e.g. participant privacy or use of data from a third party—those must be specified.

Reviewer #1: Yes

Reviewer #2: Yes

Reviewer #3: Yes

**********

5. Is the manuscript presented in an intelligible fashion and written in standard English?

PLOS ONE does not copyedit accepted manuscripts, so the language in submitted articles must be clear, correct, and unambiguous. Any typographical or grammatical errors should be corrected at revision, so please note any specific errors here.

Reviewer #1: Yes

Reviewer #2: Yes

Reviewer #3: Yes

**********

6. Review Comments to the Author

Please use the space provided to explain your answers to the questions above. You may also include additional comments for the author, including concerns about dual publication, research ethics, or publication ethics. (Please upload your review as an attachment if it exceeds 20,000 characters)

Reviewer #1: I would like to thank the authors for addressing my comments.

The manuscript has improved significantly, and now clarifies the outcome "Deaths involving Antipsychotics" and specifies how that outcome was operationalized.

Thank you

Reviewer #2: As the authors adequately revised the manuscript according to the reviewers' comments, I have no further comments and think that the current version is now acceptable.

Reviewer #3: Thank you for providing revisions to your manuscript. The remaining comments I have are:

1) I still think more could be done to establish the link between ethnic density and antipsychotic mortality. You cited literature about ethnic density protecting against psychosis but not how this translates to reduced antipsychotic-related mortality. Can you provide any evidence to support better cardiovascular and/or metabolic health in these areas that could counteract the adverse events seen with antipsychotics? If you are attributing the protective effect to "better engagement with healthcare services" then please expand on how specifically this could reduce antipsychotic mortality. You do not have to state a claim, just hypothesize as to what might be driving your finding.

2) You do a nice job discussing potential mechanisms underlying antipsychotic mortality, but I am still left to wonder why it is increasing over time. You brought up dosing- do you have evidence that dosing is increasing over time? Does this in any way explain the regional differences you observed? Same a above, you do not have to state a claim but hypothesize as to what might underly your main finding.

**********

7. PLOS authors have the option to publish the peer review history of their article (what does this mean?). If published, this will include your full peer review and any attached files.

If you choose “no”, your identity will remain anonymous but your review may still be made public.

**Do you want your identity to be public for this peer review?**  For information about this choice, including consent withdrawal, please see our Privacy Policy.

Reviewer #1: **Yes:** Mohammed A. Alarabi

Reviewer #2: No

Reviewer #3: **Yes:** Shaina Schwartz

**********

[NOTE: If reviewer comments were submitted as an attachment file, they will be attached to this email and accessible via the submission site. Please log into your account, locate the manuscript record, and check for the action link "View Attachments". If this link does not appear, there are no attachment files.]

To ensure your figures meet our technical requirements, please review our figure guidelines: https://journals.plos.org/plosone/s/figures

You may also use PLOS’s free figure tool, NAAS, to help you prepare publication quality figures: https://journals.plos.org/plosone/s/figures#loc-tools-for-figure-preparation.

NAAS will assess whether your figures meet our technical requirements by comparing each figure against our figure specifications.

# Author comment: Author response to Decision Letter 2

**DOI:** 10.1371/journal.pone.0349877.r004

26 Apr 2026

This document has been uploaded separately.

Attachment Submitted filename: Response_to_Reviewers_Comments_auresp_2.docx

# Peer review: Decision Letter 2

**DOI:** 10.1371/journal.pone.0349877.r005

**Authors:** Hikaru Hori

6 May 2026

Trends in Recorded Deaths involving Antipsychotics: The Role of Deprivation, Ethnicity, and Regional Disparities

PONE-D-25-54201R2

Dear Dr. Khan,

We’re pleased to inform you that your manuscript has been judged scientifically suitable for publication and will be formally accepted for publication once it meets all outstanding technical requirements.

Within one week, you’ll receive an e-mail detailing the required amendments. When these have been addressed, you’ll receive a formal acceptance letter and your manuscript will be scheduled for publication.

An invoice will be generated when your article is formally accepted. Please note, if your institution has a publishing partnership with PLOS and your article meets the relevant criteria, all or part of your publication costs will be covered. Please make sure your user information is up-to-date by logging into Editorial Manager at Editorial Manager® and clicking the ‘Update My Information' link at the top of the page. For questions related to billing, please contact billing support.

If your institution or institutions have a press office, please notify them about your upcoming paper to help maximize its impact. If they’ll be preparing press materials, please inform our press team as soon as possible -- no later than 48 hours after receiving the formal acceptance. Your manuscript will remain under strict press embargo until 2 pm Eastern Time on the date of publication. For more information, please contact onepress@plos.org.

Kind regards,

Hikaru Hori

Academic Editor

PLOS One

Additional Editor Comments (optional):

Reviewers' comments:

Reviewer's Responses to Questions

**Comments to the Author**

1. If the authors have adequately addressed your comments raised in a previous round of review and you feel that this manuscript is now acceptable for publication, you may indicate that here to bypass the “Comments to the Author” section, enter your conflict of interest statement in the “Confidential to Editor” section, and submit your "Accept" recommendation.

Reviewer #3: All comments have been addressed

**********

2. Is the manuscript technically sound, and do the data support the conclusions?

The manuscript must describe a technically sound piece of scientific research with data that supports the conclusions. Experiments must have been conducted rigorously, with appropriate controls, replication, and sample sizes. The conclusions must be drawn appropriately based on the data presented.

Reviewer #3: Yes

**********

3. Has the statistical analysis been performed appropriately and rigorously?

Reviewer #3: I Don't Know

**********

4. Have the authors made all data underlying the findings in their manuscript fully available?

The PLOS Data policy requires authors to make all data underlying the findings described in their manuscript fully available without restriction, with rare exception (please refer to the Data Availability Statement in the manuscript PDF file). The data should be provided as part of the manuscript or its supporting information, or deposited to a public repository. For example, in addition to summary statistics, the data points behind means, medians and variance measures should be available. If there are restrictions on publicly sharing data—e.g. participant privacy or use of data from a third party—those must be specified.

Reviewer #3: Yes

**********

5. Is the manuscript presented in an intelligible fashion and written in standard English?

PLOS ONE does not copyedit accepted manuscripts, so the language in submitted articles must be clear, correct, and unambiguous. Any typographical or grammatical errors should be corrected at revision, so please note any specific errors here.

Reviewer #3: Yes

**********

6. Review Comments to the Author

Please use the space provided to explain your answers to the questions above. You may also include additional comments for the author, including concerns about dual publication, research ethics, or publication ethics. (Please upload your review as an attachment if it exceeds 20,000 characters)

Reviewer #3: Thank you for responding to my comments and submitting revisions. I have no further recommendations.

**********

7. PLOS authors have the option to publish the peer review history of their article (what does this mean?). If published, this will include your full peer review and any attached files.

If you choose “no”, your identity will remain anonymous but your review may still be made public.

**Do you want your identity to be public for this peer review?**  For information about this choice, including consent withdrawal, please see our Privacy Policy.

Reviewer #3: **Yes:** Shaina Schwartz

**********

# Editor report: Acceptance letter

**DOI:** 10.1371/journal.pone.0349877.r006

**Authors:** Hikaru Hori

PONE-D-25-54201R2

PLOS One

Dear Dr. Khan,

I'm pleased to inform you that your manuscript has been deemed suitable for publication in PLOS One. Congratulations! Your manuscript is now being handed over to our production team.

At this stage, our production department will prepare your paper for publication. This includes ensuring the following:

* All references, tables, and figures are properly cited

* All relevant supporting information is included in the manuscript submission,

* There are no issues that prevent the paper from being properly typeset

You will receive further instructions from the production team, including instructions on how to review your proof when it is ready. Please keep in mind that we are working through a large volume of accepted articles, so please give us a few days to review your paper and let you know the next and final steps.

Lastly, if your institution or institutions have a press office, please let them know about your upcoming paper now to help maximize its impact. If they'll be preparing press materials, please inform our press team within the next 48 hours. Your manuscript will remain under strict press embargo until 2 pm Eastern Time on the date of publication. For more information, please contact onepress@plos.org.

You will receive an invoice from PLOS for your publication fee after your manuscript has reached the completed accept phase. If you receive an email requesting payment before acceptance or for any other service, this may be a phishing scheme. Learn how to identify phishing emails and protect your accounts at https://explore.plos.org/phishing.

If we can help with anything else, please email us at customercare@plos.org.

Thank you for submitting your work to PLOS ONE and supporting open access.

Kind regards,

PLOS ONE Editorial Office Staff

on behalf of

Dr. Hikaru Hori

Academic Editor

PLOS One
